# Does the MRI/fMRI Procedure Itself Confound the Results of Meditation Research? An Evaluation of Subjective and Neurophysiological Measures of TM Practitioners in a Simulated MRI Environment

**DOI:** 10.3389/fpsyg.2020.00728

**Published:** 2020-04-28

**Authors:** Frederick Travis, Jonathan Nash, Niyazi Parim, Barry H. Cohen

**Affiliations:** ^1^Center for Brain, Consciousness and Cognition, Maharishi University of Management, Fairfield, IA, United States; ^2^Retired, Chiangmai, Thailand; ^3^Mindful Education Lab, New York University Steinhardt School of Culture, Education, and Human Development, New York, NY, United States

**Keywords:** MRI, fMRI, EEG, TM, meditation

## Abstract

Early research into meditation, including Transcendental Meditation (TM), relied exclusively on EEG to measure brain activity during meditation practice. Since the advent of neural imaging, MRI, and later fMRI, have dominated this field. Unfortunately, the use of this technology rests on the questionable assumption that lying down in a confining tube while exposed to very loud sounds would not interfere with the meditation practice. The present study was designed to assess the effects of the fMRI procedure on both the subjective and neurophysiological responses of short and long-term TM practitioners. Twenty-three TM practitioners volunteered to participate in this study: 11 short-term meditators, averaging 2.2 years practice, and 12 long-term meditators, averaging 34.8 years. The repeated-measures design included two activities for each participant, eyes-closed rest, and TM practice, in each of three conditions: sitting quietly in an upright position (normal TM practice); lying quietly in a supine position; and lying, with earplugs, inside a simulated fMRI tube (simMRI), while exposed to 110 dB recordings of an actual fMRI machine. Subjective experiences were collected after each activity in each condition. Physiological arousal was recorded using skin conductance levels. Scalp EEG was averaged into eight frequency bands within frontal and parietal leads; eLORETA software was used to explore the 3-D cortical distribution of EEG sources. During the simMRI condition, participants reported having more shallow meditation experiences, and greater agitation/distraction. Skin conductance levels paralleled self-reports, decreasing least during the simMRI condition. Frontal and parietal power decreased from sitting to simMRI in the alpha2 through gamma bands. Parietal power was higher during rest compared to TM in the alpha1 through beta2 bands. Frontal and parietal alpha1 coherence were highest during the simMRI condition. The eLORETA analysis revealed that the default mode network was more active during TM when sitting compared to the simMRI condition. The responses to the supine condition were generally between sitting and simMRI, with some significant exceptions. In conclusion, these data indicate that the fMRI procedure itself (high dB noise; lying down) strongly influences subjective and neurophysiological responses during meditation practice, and may therefore confound the interpretation of results from fMRI studies.

## Introduction

With the advent of meditation research in the 1950’s, early researchers relied on electroencephalography (EEG) technology to gain insight into brain activity and mental states during meditation (Das and Gastaut, 1955; Wenger and Bagchi, 1961). EEG remained the primary investigative tool for over 40 years, with over 100 published studies (Lutz et al., 2007), until the introduction of magnetic resonance imaging (MRI) technology in the 1980’s. Beginning with the first brain mapping/scanning study of meditators by Herzog et al. (1990–1991) (using PET technology to investigate eight members of a Yoga meditation group), and continuing over the next decade with other pioneers in the field (Lou et al., 1999 using PET, Newberg et al., 2001 using SPECT), neural imaging would soon become the dominant research modality for the nascent field of contemplative neuroscience. The invention of BOLD (blood-oxygen-level dependent) contrast technology (Ogawa et al., 1990) used in functional MRI (fMRI) allowed brain mapping researchers to avoid the intravenous injection of contrasting dyes and the exposure to ionizing radiation required by PET and SPECT. Since its initial use in meditation research (Lazar et al., 2000), fMRI has become the most popular brain scanning technique in the field.

The investigative methodology and research design devised by these early meditation researchers, and others such as Lutz et al. (2007), created a model that would become standard protocol for virtually all future brain mapping meditation studies. Typically, experienced meditation lay practitioners or monks were recruited from long-standing contemplative/meditative traditions – e.g., Tibetan or Theravada Buddhism; various forms of Zen or Yoga, etc. Apparently, all of the early and subsequent MRI and fMRI studies proceeded on the rather dubious assumption that the MRI environment would not significantly affect the normative meditation experience of their subjects.

We question the assumption that the meditation practitioner’s usual environment (often seated in a dark or semi-lit, quiet room) could be equated to the noxious and sterile clinical environment of an MRI laboratory. Specifically, we question the assumption that positional changes (lying down vs. seated); being confined in a small tube; and exposure to vibrations and loud (>100 dB) pulsating sounds, would not confound the neurophysiological and subjective experiences of one’s normal meditation practice.

Concern about the likely contamination of fMRI findings due to the inherent noxious auditory environment was well documented as early as 2003 (Moelker and Pattynama, 2003). Their concerns about direct and indirect confounding effects are directly applicable to our misgivings regarding meditation research, as highlighted by the following excerpts:

*“MR-related acoustic noise may interfere with functional MR acquisitions both through direct and indirect pathways. Direct interference occurs because the acoustic noise in itself induces an increase in regional cerebral blood flow, interacting with the BOLD response of the brain activation of interest, and demonstrating significant effect in the primary and secondary auditory cortical structures. Indirect interference implies that acoustic noise may affect the perception and processing of the stimulus of interest by a distracting effect.”* (p. 125)

*“Accordingly, the changes in attention as a result of MR-related acoustic noise may lead to both an increase in activity in attention-related brain areas and to a drop in cortical activity in the brain areas of interest (distraction). The location of these effects can be appreciated at both cortical and subcortical levels.”*(p. 128)

For instance, temporo-parietal cortices become activated in the initial stages of build-up of auditory spatial representation (At et al., 2011). MRI noise is systematic, though atonal, but may lead to systematic increases in blood flow in temporo-parietal cortices. Recently, Nash and Newberg raised similar concerns about the reliance on MRI in contemplative neuroscience research, and argued that it was essential for researchers to account for positional and auditory factors that could confound the neurobiological findings of their subjects during meditation (Nash and Newberg, 2013; Newberg, 2014). However, in our review of four recent extensive meta-analyses of meditation neuroscience research we were surprised to find that none of these publications mentioned any concern about these confounding factors (Tomasino et al., 2012; Vago and Silbersweig, 2012; Fox et al., 2014; Tang et al., 2015). To the contrary, researchers have been comfortably following the same research design and methodology without attending to the likely possibility of interference from the MRI procedure itself. As such, we contend that researchers cannot be certain which of their brain-mapping data are mapping the meditation processes and experiences and which are artifacts caused by the procedures and equipment utilized.

This study begins to address these issues by utilizing subjective reporting, EEG, and electrodermal activity (EDA) technology in a controlled and carefully simulated MRI environment in an attempt to identify the potentially confounding elements of the MRI procedure. To determine whether long-term meditative experience can mitigate the simulated MRI effects, we recruited both long term (LT) and short term (ST) Transcendental Meditation practitioners from the campus of Maharishi University of Management and the surrounding community of Fairfield, Iowa to participate in this study.

### The Transcendental Meditation^®^ Technique (TM)

TM is a specific meditation technique that originated in India within the Vedic tradition and was introduced to the West in the late 1950’s by Maharishi Mahesh Yogi. The technique is made available worldwide by certified TM teachers and is taught in the U.S. through 180 Centers under the auspices of the Maharishi Foundation USA, a federally recognized 501(c)(3) non-profit educational organization.

This meditation practice involves the use of a given mantra (a particular sound that can be pronounced, but has no literal meaning to the practitioner), for 15–20 min twice per day while sitting comfortably with the eyes closed. However, unlike most mantra meditations, the TM technique does not involve concentration, such as would be needed to keep the mantra in awareness or maintain a mental rehearsal of the mantra. Rather, TM practice is a process of “effortless transcending” (Travis and Parim, 2017; Mahone et al., 2018). That is, the practitioner uses the mantra as a vehicle to facilitate the movement of attention from the ordinary thinking level to the least excited state of consciousness – consciousness without content, described as “pure consciousness” in the Bhagavad Gita, Yoga Sutra, and Upanishad (Maharishi Mahesh Yogi, 1969; Reddy et al., 1999; Egenes, 2012). One could reasonably assume that the loud noises of the MRI environment might be more disruptive to TM meditation experiences compared to other meditation practices. While most other meditation practices use mental control to shape cognitive or affective experiences (which could include ignoring environmental noise), transcending during TM (decreasing mental content) can be considered to be the exact opposite to the 110 dB sounds produced by MRI equipment. Thus, we argue that assessing the effects of a simulated MRI environment on experiences during TM practice would be a sensitive test of the possible effects of the MRI procedure on meditation experiences, in general.

Transcending is marked by distinct subjective and physiological correlates. Content analysis of descriptions of deep experience during TM practice yielded three themes – absence of time, space, and body sense (Travis and Pearson, 2000), which is the opposite of the noisy, MRI environment. Random assignment studies report that transcending is marked by reduction in sympathetic tone (Orme-Johnson, 1973; Dillbeck and Orme-Johnson, 1987) and sympathetic reactivity (Travis et al., 2009). Also, random assignment studies report increases in frontal alpha coherence and decreases in frontal beta coherence during TM practice compared to resting controls (Travis et al., 2010).

Frontal alpha1 coherence is the most consistent pattern seen during TM practice. In comparison, meditations that involve focused attention such as Vipassana or Zen are characterized by gamma power and coherence (Travis and Shear, 2010), and meditations that involve open monitoring such as mindfulness are characterized by theta and alpha2 power (Lomas et al., 2015; Travis, 2019).

The different frequency bands reflect different cortical and subcortical drivers. EEG in the delta frequency range (0.05–4 Hz) is primarily driven by reticular formation modulation; theta EEG (4–8 Hz) is primarily driven by hypothalamic-septo-hippocampal pacemakers; alpha and beta1 EEG (9–22 Hz) is primarily driven by thalamo-cortical, cortico-thalamic, and cortico-cortical oscillations; and beta 2 and gamma EEG (22–100 Hz) is primarily driven by local cortical loops involving GABA and gap junction local inhibition and excitation (Thatcher, 2016). In addition, the alpha band (8–12 Hz) has been divided into alpha1 (8–10) and alpha2 (10–12). Alpha2 activity has been seen as a marker of cortical idling, correlated with lower posterior cerebral metabolic rate (Oakes et al., 2004). Alpha1 has been correlated with restful alertness, and correlated with higher frontal cerebral metabolic activity (Mahone et al., 2018).

Skin conductance levels are not often reported during different meditation practices. However, skin conductance is a direct measure of sympathetic nervous activation and so can be useful to detect changes in physiological arousal during different conditions.

### The Present Study

The present study probes effects of an MRI environment on subjective experiences and neurophysiological measures of the sympathetic nervous system and brain activity during TM practice. The main challenges to this research were logistical – how could we effectively evaluate the effects of an MRI without access to an actual MRI machine; and even if we could, how would we measure EEG and electrodermal activity (EDA) given that we lacked the necessary technology to shield these sensitive instruments from the strong electromagnetic fields produced by an MRI machine? In order to overcome these issues, we devised a simulated MRI environment (simMRI) by building a tube of similar dimensions to an actual MRI machine, and using a recording of an actual fMRI machine played back at 110 dB through speakers strategically located near the supine subject’s head.

We compared subjective reports and neurophysiological markers during eyes closed (EC) rest and TM practice in three conditions: (1) sitting upright, the usual position to practice TM; (2) lying down in a supine position without noise; and (3) lying down in a supine position inside our simMRI as described above. Subjective reports were measured by questionnaires administered after each stage of the research; sympathetic activation was measured by skin conductance levels using an EDA recording device; and patterns of brain activity were measured by EEG power, coherence, and eLORETA activation. To determine whether more years of TM practice would mitigate the disruptive effects of the simMRI condition, we compared a group of subjects with relatively few years of experience (“Short-term” or ST) to an equal-sized group of subjects with considerably more years of experience (“Long-term” or LT).

### Primary Research Hypotheses

*Hypothesis 1*: Subjective measures of the meditative experience for both ST and LT meditators will reveal more reports of distraction or agitation, fewer reports of pure consciousness, and lower ratings of meditation depth during simMRI than the sitting quietly condition, with the supine condition falling somewhere in-between.
*Hypothesis 2*: Electrodermal activity for both ST and LT meditators will reveal more sympathetic activation during simMRI than the sitting quietly condition, with the supine condition falling somewhere in-between.
*Hypothesis 3*: Neurological measures of brain states for both ST and LT meditators (EEG power, EEG coherence, and eLORETA) will least resemble expected readings for TM during simMRI, and most resemble expected readings during the sitting quietly condition, with the supine condition falling somewhere in-between. Specifically, we expected the least frontal alpha1 power and coherence during simMRI, the most during sitting, and an intermediate amount during the supine condition.
*Hypothesis 4*: When comparing both groups, LT meditators will exhibit smaller differences in subjective, EDA, and EEG measures among the three conditions than will ST meditators, and will report less interference from the simMRI.

## Materials and Methods

### Participants

Twenty-three individuals from the meditating community in Fairfield, Iowa and the Maharishi University of Management campus volunteered for the study. The entire sample consisted of 11 ST meditators (9 males; age: *M* = 27.2 years, *SD* = 7.04; TM experience: *M* = 2.17 years, *SD* = 2.51), and 12 LT meditators (12 males; age: *M* = 59.25 years, *SD* = 8.36; TM experience: *M* = 34.75 years, *SD* = 8.90). All 23 participants contributed self-report subjective data.

Three participants had unusable EEG recordings due to excessive artifact. Thus, there were only 20 subjects in the EEG analysis (11 ST: 9 males; age: *M* = 27.2 years, *SD* = 7.04; TM experience: *M* = 2.17 years, *SD* = 2.51; and 9 LT: 9 males; age: *M* = 57.7 years, *SD* = 8.94; TM experience: *M* = 33.3 years, *SD* = 9.91).

Six participants had unusable EDA recordings due to equipment malfunction (two of those six were also among the three who had unusable EEG). Thus, there were only 17 subjects in the EDA analysis (8 ST: 6 males; age: *M* = 29.4 years, *SD* = 6.78; TM experience: *M* = 1.34 years, *SD* = 0.92; and 9 LT: 9 males; age: *M* = 57.4 years, *SD* = 8.79; TM experience: *M* = 32.9 years, *SD* = 9.40).

### Experimental Design

To maximize power, a repeated-measures design was used. Each participant was run in a single session consisting of three conditions and two activities in each condition: (a) sitting quietly in a chair with eyes closed (EC) for 3 min, followed by performing TM for 7 min; (b) lying down quietly on a cot in a supine position with EC for 3 min, followed by performing TM for 7 min; and (c) lying down on a cot during an MRI simulation (simMRI) with EC for 3 min, followed by performing TM for 7 min. To control for sequence effects between the sitting and supine conditions. these two conditions were counterbalanced, such that half the participants were run through conditions a, b, c in that order, and the other half were run in the b, a, c order. It was not considered feasible to run any condition after simMRI, so that condition was always last.

### Procedure

Participants came individually by appointment to the Center for Brain, Consciousness and Cognition at Maharishi University of Management in the early afternoon around 2:00 p.m. After completing consent and demographic forms, EEG and EDA sensors were applied. EEG and EDA were then recorded throughout all phases of the experiment. Prior to the eyes-closed periods, all participants were instructed to “close your eyes and sit easily, and do not begin your TM practice until told to do so.” After the EC period, they were verbally instructed to open their eyes and answer three yes/no questions: (1) At any time did you feel sleepy? (2) At any time did you feel unusually agitated or distracted? and (3) Did you experience moments of pure consciousness? Then participants were instructed to close their eyes again and begin a 7 min TM period.

Participants were verbally instructed to end their meditation session, open their eyes, and complete another section of the questionnaire, which included the same three questions as above, plus others specifically directed at the meditative experience (see Appendix A). When participants were lying down the questions were asked verbally by the technician, the participants dictated their responses, and the technician wrote them down on the questionnaire form; for the sitting conditions participants filled out the form by themselves. Participants completed a section of the questionnaire after each EC and TM period (6 sections in total) which helped to reduce sequence effects, and maximize an accurate recounting of experience.

#### The SimMRI Environment

The simMRI environment included: (1) a cot; (2) a custom-constructed cardboard enclosure replicating the internal dimensions of a typical MRI machine (18 inches diameter); (3) two speakers strategically placed near the participant’s head playing 110 dB sounds from an actual recording of a Siemens Echo-Planar Imaging (EPI) machine for functional scans)^1^. All participants wore generic ear plugs during this condition per usual MRI protocol.

### Data Acquisition and Analysis

#### Subjective Experience: Self-Reports

A questionnaire was devised to gather subjective data from each participant after the EC and TM periods of each condition of the experiment. The questionnaire consisted of a mix of 24 yes/no, open-ended, and five-point Likert scale questions (see Appendix A). The responses on the Likert scale items were converted to numbers and averaged across participants. If the participant responded between two anchor points, the data was recorded in half steps.

#### EDA Acquisition and Analysis

EDA was recorded using a NeuLog NUL-217 Galvanic Skin Response Logger Sensor (v.2015.6). The sensors were Ag/AgCl snap electrodes attached to the tips of the 3rd and 4th fingers of their dominant hand using TD246 skin conductance paste. Skin conductance values were recorded 100 times/sec in microsiemens over the length of each recording period. The skin conductance levels at the end of each 3-min EC rest period were subtracted from their initial levels to create EDA change scores. In addition, to facilitate comparison with the resting phase, the skin conductance level after 3 min of the TM period was subtracted from the initial value to create an intermediate change score.

#### EEG Acquisition and Analysis

The EEG was recorded with the BioSemi ActiveTwo System^2^; 32 active-sensors were applied in the 10-10 system with a forehead ground, and left and right earlobe sensors for re-referencing offline. Resistance was <10 kΩ at each sensor. All signals were digitized on line at 256 points/s, with no high or low frequency filters, and stored for later offline analyses. The EEG during all EC rest and TM practice periods were visually scanned, and any epochs with movement, electrode, or eye-movement artifacts were manually marked and not included in the spectral analyses. The artifact-free data were re-referenced to the averaged signal from the left and right earlobes, and digitally filtered with a 2–45 Hz band pass filter, and fast Fourier transformed in 2-s epochs, using a Hanning window with a 20% onset and offset. Power estimates were calculated in 0.5 Hz bins and then averaged into eight frequency bands: delta (1–4.5 Hz) theta (5–7.0 Hz), alpha1 (7.5–10.0 Hz), alpha2 (10.5–12.5 Hz), sigma (13–16 Hz), beta1 (16.5–20 Hz), beta2 (20.5–30 Hz), and gamma bands (30.5–50 Hz). Similarly, coherence estimates were calculated in 0.5 Hz bins, and averaged into the same eight frequency bands. The coherence estimates for each band were then averaged across all of the 36 possible pairs among the nine frontal sensors (AF3, AF4, F3, F4, F7, F8, Fz, FC1, FC2), the 36 possible pairs among the nine parietal sensors (PO3, PO4, P3, P4, P7, P8, Pz, CP1, CP2) and the four coherence pairs among frontal and parietal sensors (F3/P3, F4/P4, AF3/P3, AF4/P4). We primarily looked at frontal and parietal areas because they represent two different styles of processing most relevant to our study of TM meditators – abstract, symbolic and executive processing in the frontal, and sensory processing in the parietal. Frontal alpha is frequently reported during TM transcending and so is more salient to test the study hypotheses. In addition, because subjects were lying down, they were lying on the occipital sensors, which led to artifacts. We did perform some exploratory analyses on the temporal EEG, but the results were virtually identical to what we found in the parietal region. Since we had no specific expectations for the temporal EEG we did not continue to analyze the temporal data.

#### eLORETA Analysis

eLORETA was used to explore 3-D cortical distributions of sources of scalp-recorded electrical potentials in the same eight frequency bands used for coherence analysis. eLORETA (exact LORETA) was developed at the KEY Institute for Brain-Mind Research at the University of Zurich to calculate 3-D patterns of activation in known gray matter areas (Pascual-Marqui et al., 1994). While this software has low spatial resolution, as is characteristic of all EEG measurements, the eLORETA algorithms are asserted to have zero localization error (Pascual-Marqui, 2002). The current implementation of eLORETA uses a realistic head model calculated by Fuchs, Kastner, Wagner, Hawes, and Ebersole (Fuchs et al., 2002), and electrode coordinates provided by Jurcak, Tsuzuki, and Dan (Jurcak et al., 2007). We compared eLORETA activation patterns during TM practice during the three conditions – sitting, supine, and simMRI.

### Statistical Analysis

Repeated-measures ANOVAs were conducted for self-report averages, EDA levels, EEG power, and EEG coherence, using IBM SPSS statistical software (version 25). Mixed-design ANOVAs were performed whenever short-term vs. long term meditation experience was added as a between-subjects factor. Due to concerns about the sphericity assumption, Greenhouse-Geisser *df*’s and *p*-values are reported for all analyses that involve more than two levels of a repeated-measures factor. eLORETA includes statistical software to test differences in cortical activation patterns. The resulting *t* statistics were mapped onto top, sagittal, and back images of the brain. An alpha of 0.05 was used to establish statistical significance for each test; two-tailed tests were used for all two-group comparisons.

## Results

### Subjective Reports of Experience

#### Depth of Meditation Experiences

**FIGURE 1:**
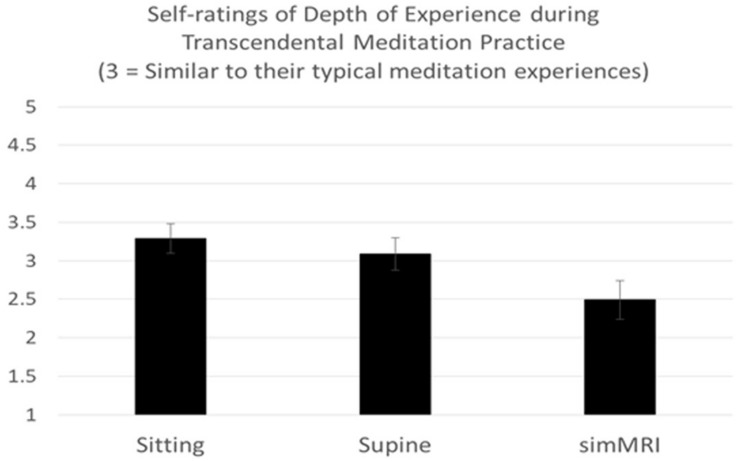
Means and standard errors of self-rating of experiences during Transcendental Meditation practice (1 = Less Deep to 5 = Deeper).

A two-way mixed-design ANOVA was conducted on the ratings of the depth of TM experience that were given after each of the three conditions, with experience (ST vs. LT) included as the between-groups factor. Only the main effect of condition was significant, *F*(1.52, 30.45) = 5.89, *p* = 0.012, η^2^*_*p*_* = 0.23. Pairwise comparisons revealed that experiences during the simulated MRI condition (simMRI) were rated across subjects as significantly less deep than either sitting, *F*(1, 22) = 12.66, *p* = 0.002, η^2^*_*p*_* = 0.37, or lying down, *F*(1, 22) = 5.51, *p* = 0.028, η^2^*_*p*_* = 0.20. Sitting did not differ significantly from lying down. These results are presented in Figure 1 (a rating of 3 indicates “as deep as usual”). There were no differences between the ST and LT groups.

#### Effects of Lying Down and the Loud MRI Sounds on Meditation Experiences

The previous self-report of more shallow meditation experiences during the simMRI condition was supported by responses on a 5-point Likert scale about the effects of lying down and loud MRI noises on experiences. The anchor points in the Likert scale ranged from 1 (minimal effect) to 5 (extreme effect). A condition by experience group mixed-design ANOVA on these ratings yielded a significant effect of condition *F*(1, 20) = 8.93, *p* = 0.007, η^2^*_*p*_* = 0.31. The mean rating for interference was 1.5 (*SD* = 0.92) for lying down, and 2.67 (*SD* = 1.72) for simMRI. There were no differences between the ST and LT groups.

#### Level of Distraction/Agitation

Reports of interference with meditation experiences were also supported by a Y/N response to agitation/distraction in each condition. A McNemar test found a significant difference between the proportion answering “yes” in the simMRI condition (14 out of 23), and the proportion answering “yes” in the sitting condition (3 out of 23), *p* = 0.003 (two-tailed, exact). The result in the supine condition was intermediate (7 out of 23), but did not differ significantly from either of the other two conditions. There were no differences between the ST and LT groups.

#### Experience of Sleepiness

Level of sleepiness was assessed with a Y/N question. A greater proportion of ST meditators (4 out of 10) reported “yes” to feeling sleepy during the sitting condition than did the LT group (0 out of 12). Using a chi square test, this group difference was significant, χ^2^ (1) = 5.87, *p* = 0.015, φ = 0.52.

#### Experience of Pure Consciousness

Along with ST participants reporting more sleepiness, they also reported fewer experiences of pure consciousness. Participants answered yes or no with respect to the experience of pure consciousness in each condition. Meditating under the simMRI condition, only 3 of the 10 ST meditators reported “yes”, whereas 10 of the 12 LT meditators reported the experience of pure consciousness at some time during their meditation session. A chi-square test revealed this difference to be significant, χ^2^(1) = 6.42, *p* = 0.011, φ = 0.54.

### Electrodermal Activity

**FIGURE 2:**
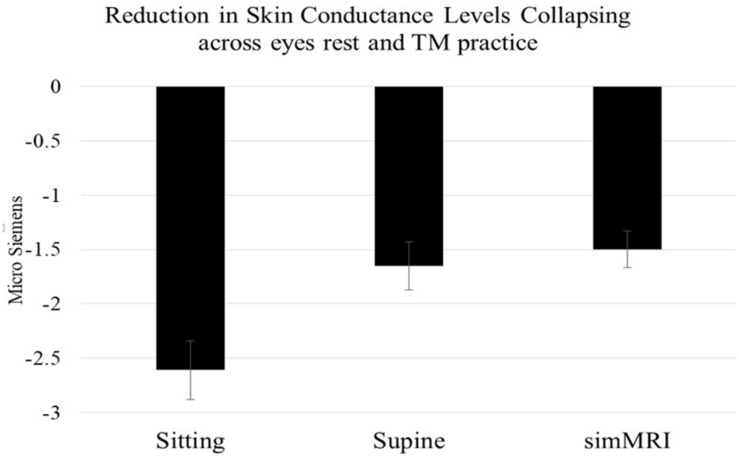
Mean and standard error EDA reductions within each condition (from beginning to 3 min into each session), collapsing across activity and experience group. Skin conductance level decreases when sitting were significantly larger than during both the supine and simMRI conditions.

A condition (sitting vs. lying vs. simMRI) by activity (EC rest vs. first 3 min of TM) by group (8 ST vs. 9 LT meditators) mixed-design ANOVA (repeated-measures on the first two factors) was conducted on the EDA change scores. Only the main effect of condition attained significance, *F*(1.86, 27.9) = 12.94, *p* < 0.001, η^2^*_*p*_* = 0.46. Pairwise comparisons revealed that the reductions in skin conductance level during the sitting condition were significantly larger than during both the supine, *F*(1, 15) = 15.5, p = 0.001, η^2^*_*p*_* = 0.51, and simMRI conditions, *F*(1, 15) = 18.3, *p* = 0.001, η^2^*_*p*_* = 0.55 (see Figure 2). The latter two conditions did not differ significantly from each other (*F* < 1). Planned comparisons found that the drop in skin conductance level during the first 3 min of TM was significantly greater than the drop during EC rest only during the sitting condition, *F*(1, 15) = 5.12, *p* = 0.039, η^2^*_*p*_* = 0.26. There were no significant differences in changes in skin conductance levels between the ST and LT groups.

### EEG Log Power: Omnibus Comparison

A condition (sitting vs. lying vs. simMRI) by activity (EC rest vs. TM) by location (frontal vs. parietal) by frequency (delta, theta, alpha1, alpha2, sigma, beta1, beta2, gamma) repeated-measures ANOVA was conducted on EEG log power. The main effects of all four factors were significant. However, there were also significant two-way interactions of condition × location, condition × frequency, and activity × location, and one three-way interaction: condition × location × frequency, *F*(4.34, 82.5) = 2.75, *p* = 0.03, η^2^*_*p*_* = 0.13. Thus, separate ANOVAs were conducted with condition, activity and frequency as repeated-measures factors, within the frontal and parietal locations.

#### Frontal Log Power

In the frontal area, the condition × frequency interaction was on the borderline of significance, *F*(2.86, 54.3) = 2.70, *p* = 0.058, η^2^*_*p*_* = 0.12. Therefore, we performed separate condition (sitting vs. lying vs. simMRI) × activity (EC rest vs. TM) repeated-measures ANOVAs for each of the eight frequency bands. There were no significant effects for delta or theta, but the main effect of condition was significant for each of the other bands, with the following *p*-values: alpha1, *p* = 0.016; alpha2, *p* = 0.018; sigma, *p* < 0.001; beta1, *p* = 0.002; beta2, *p* = 0.007; gamma, *p* = 0.01. For all of the bands from alpha2 to gamma, the pattern was essentially the same: power decreased from sitting to supine to simMRI.

**FIGURE 3:**
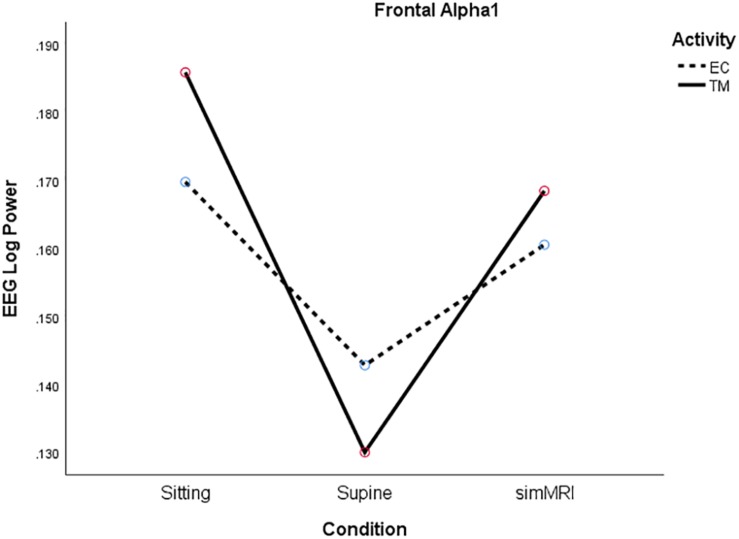
Frontal Alpha1 log power for the three conditions (sitting, supine, simMRI) and two activities: EC rest (broken), and TM (solid).

Only alpha1 exhibited a different pattern from the other frequencies. Power was lowest for the supine condition and similar for the sitting and the simMRI conditions, *F*(1.67, 31.8) = 5.15, *p* = 0.016, η^2^*_*p*_* = 0.21 (see Figure 3). Also, there was a significant main effect of activity for the beta1 band, *F*(1, 19) = 4.50, *p* = 0.047, η^2^_*p*_ = 0.19; beta1 power was higher for EC rest than TM in all three conditions.

#### Parietal Log Power

In the parietal area, significance was attained by both the condition × activity, *F*(1.99, 37.7) = 5.87, *p* = 0.006, η^2^*_*p*_* = 0.24, and frequency × activity, *F*(3.76, 71.4) = 2.81, *p* = 0.034, η^2^*_*p*_* = 0.13, interactions. Therefore, we conducted a condition × activity ANOVA for each frequency. The main effect of condition was significant for each of the bands from alpha1 to gamma (alpha1, *p* = 0.017; alpha2, *p* = 0.017; sigma, *p* < 0.001; beta1, *p* < 0.001; beta2, *p* < 0.001; gamma, *p* < 0.001), with all bands exhibiting essentially the same pattern: power decreased from sitting to supine to simMRI.

Whereas only beta1 log power was higher during rest than TM in the frontal area, there were significant activity main effects for parietal log power in five frequency bands: alpha1, *p* = 0.017; alpha2, *p* = 0.042; sigma, *p* = 0.003; beta1, *p* = 0.001; beta2, *p* = 0.027. In each case, power was higher for the EC rest phase than for TM.

### EEG Coherence: Omnibus Comparison

A condition (sitting vs. lying vs. simMRI) by activity (EC rest vs. TM) by location (frontal vs. parietal) by frequency band (delta, theta, alpha1, alpha2, sigma, beta1, beta2, gamma) repeated-measures ANOVA was conducted on EEG coherence. In addition to significant condition × activity and frequency × activity interactions, the location × condition × frequency three-way interaction was significant, *F*(5.92, 112.5) = 2.97, *p* = 0.01, η^2^*_*p*_* = 0.14. Therefore, we performed 3 (condition) by 2 (activity) by 8 (frequency) ANOVAs for each brain location.

#### Frontal Coherence

In the frontal area, significance was attained by both the condition × activity, *F*(1.85, 35.2) = 4.38, *p* = 0.022, η^2^*_*p*_* = 0.19, and frequency × activity, *F*(3.90, 74.1) = 2.54, *p* = 0.048, η^2^*_*p*_* = 0.12, interactions. Therefore, we conducted a condition × activity ANOVA for each frequency. For alpha1, only the main effect of condition was significant, *F*(1.69, 32.0) = 5.40, *p* = 0.013, η^2^*_*p*_* = 0.22. SimMRI was associated with significantly greater coherence than the supine condition, *F*(1, 19) = 11.7, *p* = 0.003, η^2^*_*p*_* = 0.38, whereas sitting was between these two extremes.

**FIGURE 4:**
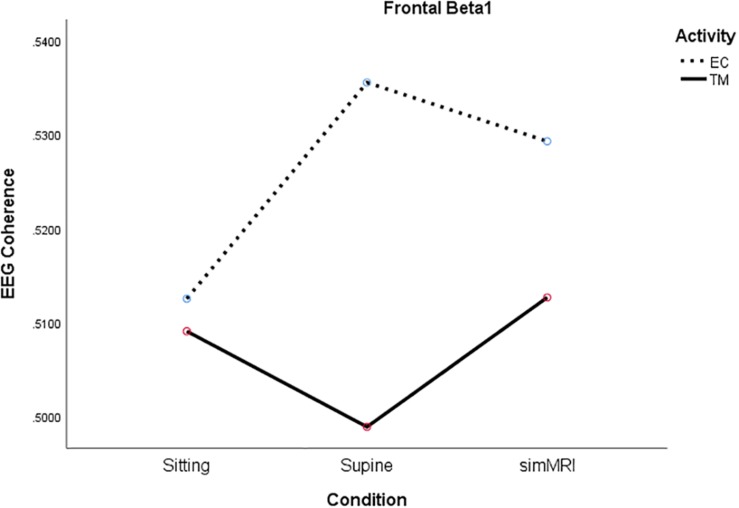
Significant condition by activity interaction for frontal beta1 coherence as a function of sitting, supine, and simMRI, during EC rest (broken) and TM practice (solid).

For beta1, the condition × activity interaction was significant, *F*(1.93, 36.6) = 5.15, *p* = 0.012, η^2^*_*p*_* = 0.21. Although the simple main effect of condition was not significant for either activity, the significant interaction is due to their opposite patterns (see Figure 4). For EC rest, the peak of coherence occurs during the supine condition; for TM, coherence is at its lowest point during the supine condition.

#### Parietal Coherence

**FIGURE 5:**
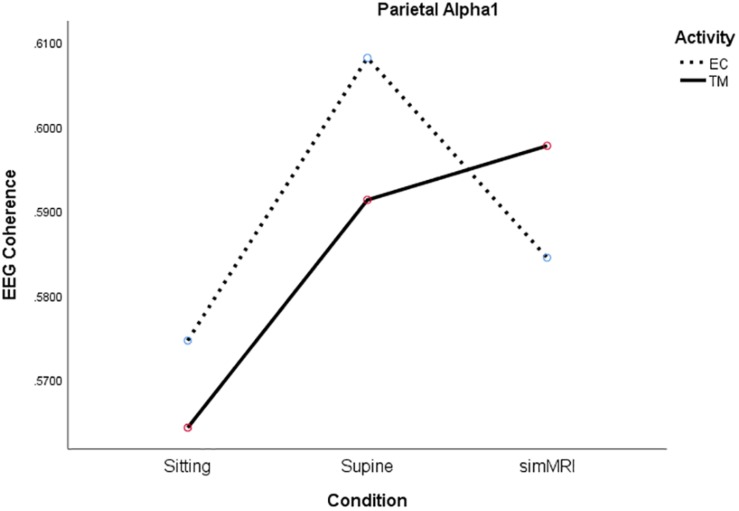
Significant condition by activity interaction for parietal alpha1 coherence as a function of sitting, supine, and simMRI, during EC rest (broken) and TM practice (solid).

In the parietal region, there was a significant condition by activity by frequency interaction, *F*(5.49, 104.4) = 2.56, *p* = 0.027, η^2^*_*p*_* = 0.12. Therefore, we performed condition × activity repeated-measures ANOVA’s separately for each frequency band. The condition × activity interactions were significant only for alpha1, *F*(1.86, 35.2) = 5.38, *p* = 0.011, η^2^*_*p*_* = 0.22, sigma, *F*(1.77, 33.6) = 4.00, *p* = 0.032, η^2^*_*p*_* = 0.17, and beta1, *F*(1.92, 36.5) = 3.80, *p* = 0.033, η^2^*_*p*_* = 0.17. As you can see from Figure 5, the interaction for alpha1 coherence occurs between the supine and simMRI conditions, such that EC rest drops from supine to simMRI, while coherence for TM continues to rise. The interaction patterns for sigma and beta1 were similar to those for alpha1.

For alpha2, only the main effect of condition attained significance, *F*(1.92, 36.5) = 4.38, *p* = 0.021, η^2^*_*p*_* = 0.19. Coherence was highest for simMRI and lowest for sitting.

#### Frontal-Parietal Coherence

The coherences of each frontal lead paired with each corresponding parietal lead were averaged together to create a measure of frontal-parietal coherence, which was then submitted to the three-way ANOVA used for the separate frontal and parietal analyses. Because there was a significant condition × frequency interaction, *F*(5.01, 95.1) = 2.37, *p* = 0.045, η^2^*_*p*_* = 0.11, we performed condition by activity ANOVAs for each frequency band. The two-way interaction was not significant at any frequency, but the main effect of activity was significant for alpha2, beta1, and beta2, with *p*-values of 0.032, 0.039, and.022, respectively. In each case, coherence was higher for EC rest than TM for all three conditions.

The main effect of condition was significant only for the sigma band, *F*(1.73, 32.9) = 4.50, *p* = 0.023, η^2^*_*p*_* = 0.19. Coherence was significantly lower for sitting than for both the simMRI and supine conditions; sigma coherence was similar for the latter two conditions. There were no significant effects for delta, theta, alpha1, or gamma.

### EEG: Comparison of Experience Groups

To address Hypothesis 4, experience group (11 ST vs. 9 LT meditators) was added as a between-groups factor to the ANOVAs performed on EEG log power and EEG coherence. A few interpretable group effects attained statistical significance.

#### Log Power

**FIGURE 6:**
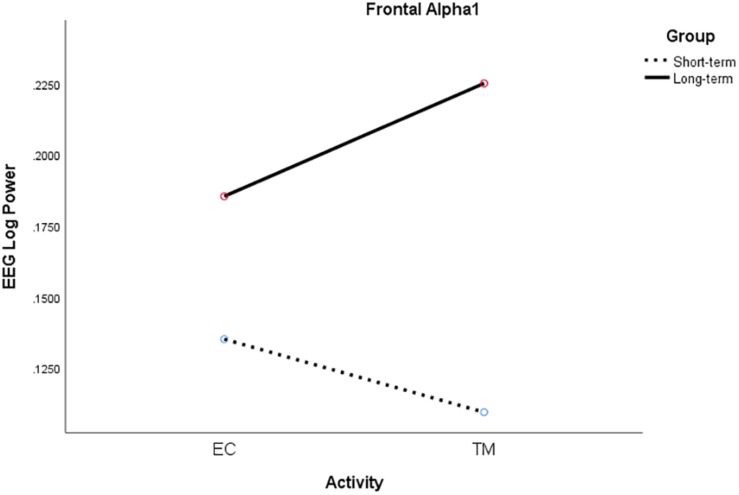
Significant activity × group interaction for frontal alpha1 log power for ST (broken) and LT (solid) meditators during EC rest and TM practice, collapsing across all conditions.

For frontal alpha1 power, there was a significant two-way interaction between activity and group, *F*(1, 18) = 4.71, *p* = 0.044, η^2^*_*p*_* = 0.21. Alpha1 power increased from EC rest to TM for LT meditators, collapsing across conditions, but decreased for ST meditators (see Figure 6).

#### Frontal Coherence

The condition by activity by group interaction was significant for the theta band, *F*(1.99, 35.8) = 4.79, *p* = 0.015, η^2^*_*p*_* = 0.21. The pattern for the LT meditators was simple: For both EC rest and TM, coherence was higher for supine than sitting, and higher still for simMRI. By contrast, the ST meditators exhibited an uninterpretable condition by activity interaction.

#### Parietal Coherence

**FIGURE 7:**
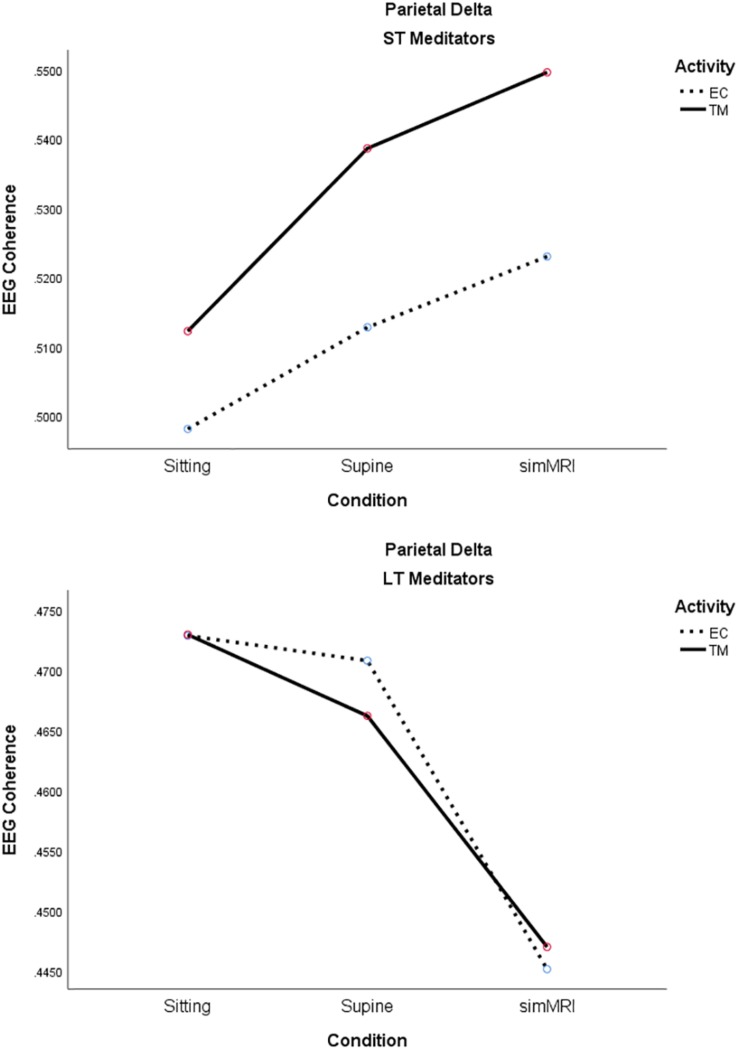
Significant effects for parietal delta coherence involving the condition, activity, and group factors (see text for details).

The main effect of experience group was significant, as well as interpretable, for the delta band, *F*(1, 18) = 6.12, *p* = 0.024, η^2^*_*p*_* = 0.25; coherence was consistently higher for ST than LT meditators during both TM and EC rest for all conditions. Moreover, group interacted significantly with condition, *F*(1.56, 28.1) = 7.40, *p* = 0.005, η^2^*_*p*_* = 0.29; it is easy to see from Figure 7 that the two groups exhibited opposite patterns with respect to condition. Finally, there was also a significant group by activity interaction, *F*(1, 18) = 8.32, *p* = 0.010, η^2^*_*p*_* = 0.32. The simple main effect of activity was significant for the ST meditators, such that their coherence was higher for TM than EC rest in all conditions, whereas coherence differed very little between the two activities for LT meditators, regardless of condition (see Figure 7).

### eLORETA

**FIGURE 8:**
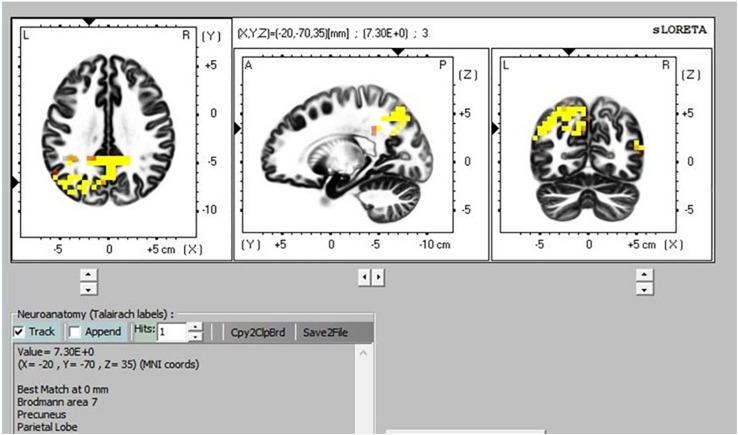
Transcendental Meditation during sitting (yellow) vs. supine.

**FIGURE 9:**
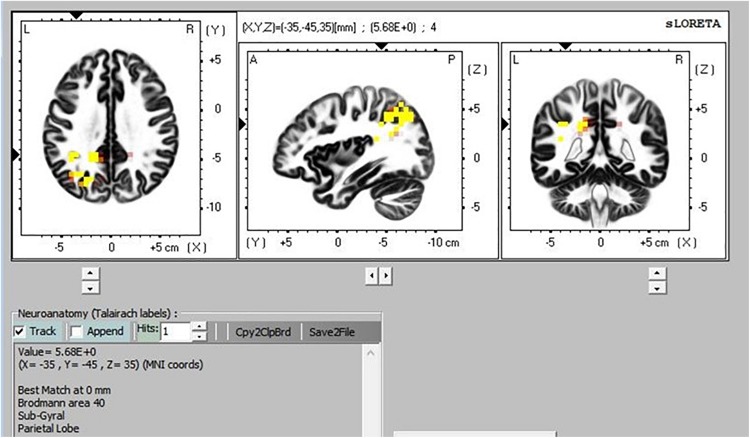
Transcendental Meditation during sitting (yellow) vs. simMRI.

There was greater activation in the precuneus part of the default mode network (DMN) during TM practice when we compared sitting to supine (Figure 8), and sitting to simMRI (Figure 9). All activation areas are significant at the *p* < 0.05 level.

## Discussion

This research asked whether the MRI procedure confounds the findings of meditation research. The self-report, skin conductance, and EEG power and coherence measures clearly indicate that the answer to this question is a resounding “Yes.”

### Effects on Depth of Meditation

Subjective meditation experiences were definitely influenced by the simMRI condition. The subjects rated progressively less deep meditation experiences, greater interference effects, and greater agitation and distraction when going from sitting to supine to the simMRI condition.

These subjective reports were supported by the skin conductance patterns, which are a direct measure of sympathetic activation levels. In this research, skin conduction levels decreased more during the sitting condition compared to the supine and simMRI condition, collapsing across the two activities – EC rest and TM practice.

### EEG Power and Coherence Paint a Complex Picture

For most frequency bands, in both regions, power patterns significantly decreased from sitting to supine to simMRI. Research on effects of posture changes on EEG report that high frequency EEG decreases when an individual moves from upright to supine (Thibault et al., 2014). This effect is thought to arise from the brain shifting in the skull from upright to supine and the resulting small changes in CSF layer thickness. The loud noises of simMRI had a further suppressing effect on EEG power, except for the alpha1 band in the frontal region, which exhibited a large, and unexpected, power increase during simMRI relative to the supine condition; simMRI power was almost as high as it was in the sitting condition. This anomalous result may represent some kind of general alerting effect of the noise. Given the prominence of frontal alpha1 power results in previous studies of meditation, this result could be a source of some concern for fMRI studies.

Frontal alpha1 coherence and parietal alpha1, sigma, and beta1 coherence were highest during simMRI compared to supine and sitting, which was not expected. The sounds of the fMRI recording are not melodious but they are systematic – clanging and banging in sequences. This systematic auditory input could drive the auditory centers in the temporal and the inferior parietal cortices which integrate input from the sensory modalities. Perhaps, these coherence patterns were more related to the effects of processing the audio-disruptive 110 dB sounds (Moelker and Pattynama, 2003) than the expected effects of meditation practice. Furthermore, the significant interactions between condition and activity for both frontal beta1 and parietal alpha1 (see Figures 4, 5) suggest that the MRI noise differentially affected the eyes-closed rest and TM phases. Together these data suggest that the simMRI condition adds systematic patterns of EEG that are not part of typical meditation EEG patterns.

### eLORETA Patterns

The eLORETA, which gives cortical sources of scalp-recorded EEG, reported higher activity in particular brain areas during TM practice when sitting compared to the TM sessions during the supine and simMRI conditions. The areas of greater activation were in the precuneus area, which is the major posterior hub of the default mode network (DMN). This network is more active when the mind is engaged in undirected thought and is deactivated when attention focuses on a task (Raichle et al., 2001). Higher activation in the DMN has been theorized as an indicator of the effortless/non-concentrative nature of TM practice (Travis and Parim, 2017). Higher DMN activation when sitting suggests that the meditation practice was more normative/transcendent when compared to the supine and simMRI conditions.

### Effects on Short-Term and Long-Term Meditators

In general, the data did not support our hypothesis that the LT meditators would exhibit smaller differences in all measures than the ST group among the three conditions. However, the data did partially affirm that LT meditators were less affected by the simMRI condition than the ST meditators. Some significant differences were seen:

Frontal alpha1 power increased from EC to TM for the LT meditators but decreased for the ST meditators, which is consistent with the ST self-reports of increased sleepiness during their meditation practice. They also exhibited higher parietal delta coherence which is consistent with greater sleepiness. On the other hand, the LT meditators reported increased experiences of pure consciousness, which is consistent with higher alpha1 power across all conditions in this group. The LT group also exhibited higher theta coherence during simMRI compared to supine and sitting. Theta reflects attention to ongoing mental activity. Longer experience with TM seemed to mitigate some of the challenges and distractions of meditating in a MRI environment.

As mentioned in Hypothesis 4, one of our primary objectives was to evaluate whether the amount of meditation experience moderates the effects of high dB audio-disruption and lying down in a supine position on our dependent variables. However, our short-term meditators, with a mean of just over 2 years of experience, were considerably younger (about 27 years old on average) than the long-term meditators, with a mean of more than 34 years of TM experience, and an average age of ∼59. Unfortunately, within the meditating community in Fairfield, Iowa we were unable to find older individuals with short-term meditation experience. Therefore, in order to conduct the research in our locale, we had to accept a disparity in age between the two groups.

This disparity in ages is a factor that could have affected some of the differences seen between these two groups. Notably, global coherence (1–30 Hz) is reported to decrease with age, along with a slowing of peak frequency (ratio of 1–8 Hz power/9–30 Hz power (Laptinskaya et al., 2019). However, this general age effect cannot easily explain the results we reported in this study. We found interactions of group with the condition factor on both EEG power and coherence, rather than an overall decrease in coherence, or in slowing of peak frequency of the EEG.

## Limitations and Suggestions for Future Research

One limitation of this study is that our simulated MRI condition may not have been as severe as the actual MRI environment. First, our simulation did not include the powerful electromagnetic fields associated with the typical MRI machine, which may, or may not, affect the meditative state and/or the ability of the practitioner to transcend. Second, none of the subjects said that they felt claustrophobic. Since this is a common reaction to being inside a real MRI machine, this calls into question whether our simulation was a good enough replica of an actual MRI environment (although it may well be possible that meditators are less phobic than the general population).

A second limitation is that our findings cannot be generalized toward all forms of meditation practice. Our study only used participants practicing the TM technique. This technique does not use mental or attention control, but allows the mind to take its natural direction. The effects of the MRI environment may differ for practitioners of other meditation techniques that involve active mental processes, such as concentration or the recitation of a particular narrative. Future research can investigate this possibility by comparing individuals practicing other forms of meditation which are inherently different from TM, such as techniques which employ concentration, loving kindness and compassion, or non-focused methods such as open monitoring and open presence. These studies should include a control group of non-meditators at eyes-closed rest in the sitting, supine, and simMRI conditions.

Future meditation research could utilize neuroimaging technology that allows for simultaneous EEG and ANS recordings inside an active MRI tunnel in order to better differentiate targeted brain activity from the confounding influences of the fMRI/MRI process. Future research could also consider recent technology that allows the patient to sit during the MRI recording (thus eliminating the confound of lying down), and equipment that produces reduced acoustical noise (such as Silenz pulse developed by GE Healthcare). This innovation reduces the scanning sound level to 69 dB which is similar to a typical vacuum cleaner. With standard ear protection equipment, the noise levels could be substantially reduced, and possibly eliminate this confound as well.

Third, it should be noted that there was a total of 23 subjects in the study and only 10 participants per experience sub-group, which is a small sample size, with insufficient power to detect even medium-sized effects between the groups. Replication with larger samples, matched for age, would provide a better test of between-group differences based on length of meditation experience, and could yield greater confidence in our conclusions.

Last, other measures of autonomic functioning, such as breath and heart rate variability, could be used to more closely assess how the MRI environment affects autonomic system functioning.

## Conclusion

Our findings call into question the assumption that research which is based on subjects meditating in a supine position during an fMRI/MRI procedure is truly representative of those subjects’ normative experience. Specifically, our data highlight the real possibility that the neural imaging process itself (high dB noise, lying down, and possible claustrophobia) may noticeably influence the neurophysiological activity being measured and thereby confound the results of the study. Under these circumstances, we question how researchers can be confident that they are looking at brain activity normally associated with meditation under non-laboratory conditions.

In addition, we think it is important to consider that the fMRI noise itself could evoke systematic brain activity that is independent of, and could be confused with, the normative meditation condition. The significant increase in frontal alpha1 coherence during the simMRI condition as compared to the quiet sitting condition is consistent with this second inference.

With regards to the practitioner experience factor, this study found some significant differences between LT and ST meditators. This demonstrates the importance of accounting for the length of meditation experience when interpreting one’s results and not assuming that a group of practitioners with varying experience can produce homogeneous findings.

Finally, given that there is a dearth of research on the effects of the fMRI/MRI procedure itself on brain activity during meditation, this study is valuable not so much for whether it confirms or disputes our initial hypotheses, but rather because it raises serious red flags for meditation researchers who might consider relying solely on neuroimaging data acquisition. Most importantly, we strongly suggest that future neuroimaging experimental protocols include an EEG component and physiological measures in order to isolate and identify possible confounding effects of the process itself, or MRI equipment that has reduced noise, and allows subjects to be scanned in a sitting position.

## Data Availability Statement

The datasets generated for this study are available on request to the corresponding author.

## Ethics Statement

The studies involving human participants were reviewed and approved by the Institutional Review Board, Maharishi International University, Fairfield, IA 52557. The patients/participants provided their written informed consent to participate in this study.

## Author Contributions

JN, FT, and NP contributed to the conception and design of the study and the subjective questionnaire. NP and FT organized the database. NP scheduled subjects, collected data, and analyzed data. BC and FT performed the statistical analysis and the construction of all figures and graphs. FT and JN wrote the first drafts of the manuscript. FT, JN, and BC wrote all subsequent additions and revisions of the manuscript. All authors contributed to the proofing of the manuscript, and read and approved the submitted version.

## Conflict of Interest

The authors declare that the research was conducted in the absence of any commercial or financial relationships that could be construed as a potential conflict of interest.

## Footnotes

1 https://www.youtube.com/watch?v=6Aj2QspPf7s

2 www.Biosemi.com
