# Supplementary material for: Does the MRI/fMRI Procedure Itself Confound the Results of Meditation Research? An Evaluation of Subjective and Neurophysiological Measures of TM Practitioners in a Simulated MRI Environment
Source: Front Psychol. 2020 Apr 28;11:728. doi: 10.3389/fpsyg.2020.00728 (PMC7198852; doi:10.3389/fpsyg.2020.00728)
Supplement: Supplementary file 1 [file Data_Sheet_1.pdf]

Appendix 'A'

AFTER CONDITION 1, PHASE 1 (Sitting: Eyes Closed)

Subject Number ______________________________ Date ___________

1. At any time during the EEG recording did you feel sleepy? Y/N

   If YES, please explain: _______________________________________

   ____

2. At any time during the EEG recording did you feel unusually agitated or distracted? Y/N

   If YES, please explain: ______________________________________

   ____

   ____

3. Please describe your experiences during the EEG recording. Did you experience moments of "pure consciousness"? Y/N

   ____

   ____

AFTER CONDITION I, PHASE 2 (Sitting: Meditation)

1. At any time during the EEG recording did you feel sleepy? Y/N

If YES, please explain: _______________________________________

____

2. At any time during the EEG recording did you feel unusually agitated or distracted? Y/N

If YES, please explain: ____

____

____

3. Please describe your experiences during the EEG recording. Did you experience moments of “pure consciousness”? Y/N

____

____

4. Please rate your meditation experience during this phase compared to your typical meditation session

/_______/_______/_______/_______/

1 3 5

Less Deep Same Deeper

AFTER CONDITION 2, PHASE 1 (Supine: Eyes Closed)

1. At any time during the EEG recording did you feel sleepy? Y/N
   If YES, please explain: ____
   ____

2. At any time during the EEG recording did you feel unusually agitated or distracted? Y/N
   If YES, please explain: ____
   ____
   ____

3. Please describe your experiences during the EEG recording. Did you experience moments of “pure consciousness”? Y/N

____

____

AFTER CONDITION 2, PHASE 2 (Supine: Meditation)

1. At any time during the EEG recording did you feel sleepy? Y/N
   If YES, please explain: ____
   ____

2. At any time during the EEG recording did you feel unusually agitated or distracted? Y/N
   If YES, please explain: ____
   ____
   ____

3. Please describe your experiences during the EEG recording. Did you experience moments of "pure consciousness"? Y/N
   ____
   ____

4. Please rate your meditation experience during this phase compared to your typical meditation session

   /_______/_______/_______/_______/

   1 3 5

   Less Deep Same Deeper

5. Did lying down interfere with your ability to meditate?

   /_______/_______/_______/_______/

   minimally extremely

AFTER CONDITION 3, PHASE 1 (simMRI: Eyes Closed)

1. At any time during the EEG recording did you feel sleepy? Y/N
   If YES, please explain: ____
   ____

2. At any time during the EEG recording did you feel unusually agitated or distracted? Y/N
   If YES, please explain: ____
   ____
   ____

3. Please describe your experiences during the EEG recording. Did you experience moments of "pure consciousness"? Y/N
   ____
   ____

AFTER CONDITION 3, PHASE 2 (simMRI: Meditation)

1. At any time during the EEG recording did you feel sleepy? Y/N
   If YES, please explain: ____
   ____

2. At any time during the EEG recording did you feel unusually agitated or distracted? Y/N
   If YES, please explain: ____
   ____
   ____

3. Please describe your experiences during the EEG recording. Did you experience moments of "pure consciousness"? Y/N
   ____
   ____

4. Please rate your meditation experience during this phase compared to your typical meditation session

/_______/_______/_______/_______/

1 3 5

Less Deep Same Deeper

5. Did your experience in the simulated MRI tube create any feeling of claustrophobia?

/________/________/________/________/

Minimally Extremely

6. Did the loud noises in the MRI tube interfere with your ability to meditate

/________/________/________/________/

Minimally Extremely
